# Examining the Species-Specificity of Rhesus Macaque Cytomegalovirus (RhCMV) in Cynomolgus Macaques

**DOI:** 10.1371/journal.pone.0121339

**Published:** 2015-03-30

**Authors:** Angie K. Marsh, Aruna P. Ambagala, Catia T. Perciani, Justen N. Hoffman Russell, Jacqueline K. Chan, Michelle Janes, Joseph M. Antony, Richard Pilon, Paul Sandstrom, David O. Willer, Kelly S. MacDonald

**Affiliations:** 1 Department of Immunology, University of Toronto, Toronto, ON, Canada; 2 Department of Microbiology, Mount Sinai Hospital, Toronto, ON, Canada; 3 National HIV & Retrovirology Laboratories, National HIV and Retrovirology Laboratories, Public Health Agency of Canada, Ottawa, ON, Canada; 4 Department of Medicine, University of Toronto, Toronto, ON, Canada; Emory University School of Medicine, UNITED STATES

## Abstract

Cytomegalovirus (CMV) is a highly species-specific virus that has co-evolved with its host over millions of years and thus restricting cross-species infection. To examine the extent to which host restriction may prevent cross-species research between closely related non-human primates, we evaluated experimental infection of cynomolgus macaques with a recombinant rhesus macaque-derived CMV (RhCMV-eGFP). Twelve cynomolgus macaques were randomly allocated to three groups: one experimental group (RhCMV-eGFP) and two control groups (UV-inactivated RhCMV-eGFP or media alone). The animals were given two subcutaneous inoculations at week 0 and week 8, and a subset of animals received an intravenous inoculation at week 23. No overt clinical or haematological changes were observed and PBMCs isolated from RhCMV-eGFP inoculated animals had comparable eGFP- and IE-1-specific cellular responses to the control animals. Following inoculation with RhCMV-eGFP, we were unable to detect evidence of infection in any blood or tissue samples up to 4 years post-inoculation, using sensitive viral co-culture, qPCR, and Western blot assays. Co-culture of urine and saliva samples demonstrated the presence of endogenous cynomolgus CMV (CyCMV) cytopathic effect, however no concomitant eGFP expression was observed. The absence of detectable RhCMV-eGFP suggests that the CyCMV-seropositive cynomolgus macaques were not productively infected with RhCMV-eGFP under these inoculation conditions. In a continued effort to develop CMV as a viral vector for an HIV/SIV vaccine, these studies demonstrate that CMV is highly restricted to its host species and can be highly affected by laboratory cell culture. Consideration of the differences between lab-adapted and primary viruses with respect to species range and cell tropism should be a priority in evaluating CMV as vaccine vector for HIV or other pathogens at the preclinical development stage.

## Introduction

Mammalian herpesviruses are ancient DNA viruses that have evolved over the last 200 million years [1]. These viruses are large double-stranded DNA viruses that establish lifelong infection in their host and are categorized into three subfamilies, alpha- beta- and gamma herpesviruses. The betaherpesvirus family, consisting of cytomegalovirus (CMV), HHV-6 and HHV-7, diverged over 170 million years ago with the subsequent divergence of CMV occurring 60 million years thereafter [1,2]. CMV is a ubiquitous pathogen that causes asymptomatic infection in immunocompetent hosts and elicits a robust CMV-specific T-cell response [3–5]. However, this strong cellular immune response is not sufficient to prevent re-infection with a different strain of CMV, as superinfection with multiple strains of human CMV (HCMV) can occur in both immunocompetent [6–8] and immunocompromised adults [9–12], as well as in children [13]. Similarly, other human herpesviruses, such as herpes simplex virus (HSV) type 1 and 2, varicella zoster virus (HHV-3), Epstein-Barr virus (HHV-4), and Kaposi's sarcoma-associated herpesvirus (HHV-8) have been shown to have a similar capacity for super-infection [14–18].

Cytomegaloviruses have co-evolved with their host species following co-speciation over 80 million years ago resulting in highly species-specific viruses [1]. This continued virus-host co-evolution has resulted in the acquisition of a series of host-specific genes, which benefit the virus. A number of these genes have augmented viral replication capacity; and the acquisition of anti-apoptotic and immune-evasion genes appear to evade the host immune response preventing complete elimination of the virus during primary infection [19]. In addition to human CMV [20–24], cytomegaloviruses have been isolated and characterized from a number of different non-human primate (NHP) species, including but not limited to, chimpanzees [25,26], rhesus macaques [27,28], cynomolgus macaques [29,30] as well as more distant species involving guinea pigs [31,32], mice [33], rats [34], and tree shrews [35], among others. It has been shown that certain non-human primate CMV strains can overcome the species-specific barrier in vitro. Cynomolgus macaque CMV-Ottawa strain (CyCMV_Ott_), rhesus macaque CMV (RhCMV), and baboon CMV (BaCMV), all have the capacity to productively infect various human cell lines [36] [29,30] [37] [38]. Furthermore, our group has demonstrated that lab-adapted CyCMV_Ott_ can productively infect rhesus macaque fibroblast cells [29,30] and likewise, RhCMV can infect cynomolgus macaque fibroblast cells [[37], Ambagala, et al. unpublished]. CyCMV_Ott_ was originally isolated from a NHP breeding colony of cynomolgus macaques of Indo-Filipino origin [30]. While RhCMV and CyCMV_Ott_ are closely related (89% nucleotide identity) at the genomic level [30], the specific genes that impact host-restriction as well as the degree of homology required to overcome species-specificity has not been investigated.

It is generally accepted that CMV species-specificity is highly restricted in vivo [39,40], though to what extent has not been well documented. Whether cross-species infection can occur between closely related species, such as Old World monkeys, remains to be elucidated. In an effort to further investigate the degree of CMV species-specificity and to determine whether cynomolgus macaques can be infected with RhCMV and used as an adjunct NHP model to evaluate RhCMV-vectored HIV/SIV vaccines, we constructed a recombinant RhCMV constitutively expressing enhanced green fluorescence protein (eGFP). The ability of this replication-competent RhCMV-eGFP to express the exogenous antigen (eGFP) enabled differentiation between productive RhCMV-eGFP infection and pre-existing CyCMV infection in CyCMV strain-seropositive cynomolgus macaques. In this study, we inoculated cynomolgus macaques with RhCMV-eGFP in an effort to establish productive infection, marked by evidence of RhCMV-eGFP-specific immune responses and detectable RhCMV-eGFP in the tissues and/or being shed in the urine. Using a wide variety of viral detection (viral co-culture, qPCR, Western blot) and immune response assays (FACS, ELISA) to extensively examine numerous tissues (blood, urine, BAL, saliva, lymph nodes, oral and genital swabs) over the course of 4 years post-inoculation, our results strongly suggest that RhCMV-eGFP was not able to infect cynomolgus macaques and the species-specific barrier could not be broken between these closely related non-human primates.

## Methods

### Monkeys

Twelve healthy adult male (range 6.78 kg–10.07 kg; mean 8.3 kg and ranging in age from 3–12 years) cynomolgus macaques (Macaca fascicularis) of Mauritius Island origin were housed in the NHP facility at Health Canada, Ottawa, Canada. All animal sampling was for research purposes and this specific study was reviewed and approved by the Health Canada Institutional Animal Care and Use Committee (IACUC), (permit #2008–035), which met the ethical, scientific and social responsibility criteria set out by the Canadian Council on Animal Care. Animals were housed in large single cages (dimensions: 33.0” x 32.5” x 39.75”) exceeding the minimum requirements with areas of both privacy and visual as well as social interaction with other animals. Specifically, the enclosure included a visible area-perch for social interactions as well as areas for privacy from other animals. They were given access to exercise runs daily for at least five days a week. These exercise runs contained structural enrichment devices such as flexible climbing material, perches, swings and rolling barrels. Other enrichment included foraging balls, puzzle feeding devices and play toys that were randomly rotated. In addition to the basic ration of monkey chow, animals were given access to a number of other foodstuffs (bananas, apples, carrots, peanuts, raisins, sunflower seeds, and commercially purchased nonhuman primate treats (i.e. PrimaTreats), which were added to the foraging devices) to add variety to their diet and to encourage them to forage. In addition, music and videos were provided in each room housing the animals.

Veterinary staff monitored the animals daily for food intake, stool consistency, and general welfare. Attention to behavioural and laboratory markers (lymphocyte counts) of stress was monitored, as well as evidence of local or systemic opportunistic disease. Any abnormal observation, including loss of appetite for greater than 24 hours, found during regular clinical evaluations or during clinical evaluations at times of sampling, was brought to the attention of the responsible veterinarian who carefully monitored the animal over the next 24–48 hours. If judged necessary, a full physical exam as well as appropriate clinical work up (hematology, immunophenotyping and biochemistry) was performed. Any clinical and pathological results were discussed with the investigators and a decision to treat and/or euthanize the animal in a timely manner was made. The decision to euthanize an animal was always to ensure the animal would not suffer, and at no time was death considered an acceptable endpoint. Animals were anesthetized with ketamine (10 mg/kg) during all inoculations and sampling procedures. Any abnormal observations found during the regular clinical evaluations were brought to the attention of veterinary staff. The animals were randomly assigned into three groups (one experimental group and two control groups): RhCMV-eGFP group (n = 6; 7M, 8M, 9M, 10M, 11M, 12M), UV-inactivated RhCMV-eGFP control group (n = 2; 4M, 5M), and media control group (n = 4; 1M, 2M, 3M, 6M). Cynomolgus macaque CMV serostatus was confirmed by ELISA prior to the study.

### Virus

Self-excisable, full-length rhesus cytomegalovirus 68–1 bacterial artificial chromosome (RhCMV-BAC) [41] was transfected into telomerase-immortalized rhesus fibroblasts (Telo-RF) to generate wild-type RhCMV 68–1 virions. RhCMV-BAC and Telo-RF cell line [42] were obtained from Dr. Peter Barry (University of California, Davis). RhCMV was propagated and titrated in Telo-RF cells using standard plaque assays. Enhanced green fluorescent protein (eGFP) was inserted into the RhCMV 68–1 genome by homologous recombination, as described previously [43]. Briefly, the multiple cloning site [21] of the pEGFP-C1 plasmid (Clontech) was excised by *BamH*I/*Bgl*II digestion to generate pEGFP-C1ΔMCS. The eGFP cassette, flanked by the CMV IE promoter and the SV40 PolyA signal, was amplified from a pEGFP-C1ΔMCS by PCR using oligonucleotides containing terminal *Asc*I restriction sites. To introduce the eGFP cassette, an *Avr*II restriction site located in the non-coding region between open reading frames (ORFs) Rh184 and Rh185 was targeted [27]. Using high fidelity PCR, a 2 kb RhCMV 68–1 genomic sequence flanking the *Avr*II restriction site was amplified and cloned into pCR-Blunt II TOPO cloning vector (Invitrogen). With an oligonucleotide linker, the *Avr*II restriction site was altered to generate a unique *Asc*I restriction site into which the eGFP cassette amplified from pEGFP-C1ΔMSC was sub-cloned. The resulting plasmid, pCR-TOPO RhCMV-eGFP, was linearized using *Not*I and transfected into Telo-RF cells using Lipofectamine 2000 (Invitrogen). Twenty-four hours post-transfection, the transfected cells were infected with BAC-derived RhCMV 68–1 at 0.1 multiplicity of infection (MOI). When the cells demonstrated 100% cytopathic effect (CPE), the cells and culture supernatant were harvested, freeze-thawed three times, and the clarified supernatant was screened for RhCMV 68-1-expressing eGFP (RhCMV-eGFP) by limiting dilution plaque assay. The plaques expressing eGFP were harvested and subjected to multiple rounds of plaque purification to obtain a purified RhCMV-eGFP virus stock. The virus stock was concentrated by overlaying the clarified viral supernatant on a sorbitol buffer (20% D-sorbitol, 50 mM Tris-Cl, 1 mM MgCl_2_) and centrifuging at 53,000 x *g* for 90 minutes at room temperature.

eGFP expression in RhCMV-eGFP virus stocks was visually confirmed by microscopy and protein expression was confirmed by Western blot assay. UV-inactivated RhCMV-eGFP was prepared by exposing the live virus to 360,000 microjoules/cm^2^ of irradiation in a UV Stratalinker 2400 (Stratagene). Following UV-inactivation, sodium pyruvate was added to a final concentration of 5 mM to neutralize any peroxide/superoxides produced during UV-inactivation. Two UV-inactivation treatments were performed to completely inactivate the virus, as confirmed by screening for CMV CPE and eGFP expression in Telo-RF cells inoculated with UV-inactivated RhCMV-eGFP.

### Inoculations

**Fig 1:**
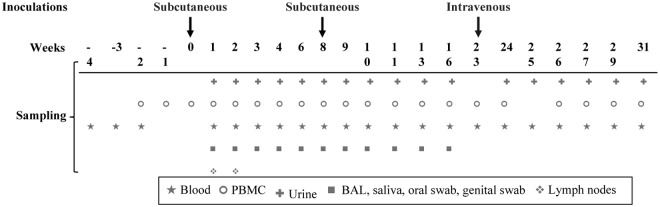
Inoculation and sampling schedule. Twelve cynomolgus macaques were randomly assigned into three groups, RhCMV-eGFP (N = 6), UV-inactivated RhCMV-eGFP control (N = 2), and media control (N = 4). The animals received one subcutaneous inoculation at week 0 with 7x10^7^ PFU of RhCMV-eGFP or UV-inactivated RhCMV-eGFP, or media alone. A subset of animals was subcutaneously boosted at week 8 with 2x10^7^ PFU of RhCMV-eGFP or UV-inactivated RhCMV-eGFP, while the remaining animals received media alone (Table 1). At week 23, one UV-inactivated RhCMV-eGFP control animal (4M) and three media control animals (1M, 2M, 6M) received an intravenous inoculation with RhCMV-eGFP (7x10^7^ PFU). Sample collection schedule is described.

Two subcutaneous inoculations were given at weeks 0 and 8, respectively, (Fig. 1; Table 1) in which the animals received 2x1 ml subcutaneous injections at different sites in the same leg. For the first inoculation, the RhCMV-eGFP inoculated animals received 7x10^7^ PFU RhCMV-eGFP cell-free virus (in DMEM+10% FBS and 0.2M sucrose) and the UV-inactivated RhCMV-eGFP control animals were given 7x10^7^ PFU UV-inactivated cell-free RhCMV-eGFP (in DMEM+10% FBS and 0.2M sucrose). The media control animals received media alone (DMEM+10% FBS and 0.2M sucrose). A second subcutaneous inoculation was given as a boost at week 8 (Fig. 1) in a subset of animals, as described in Table 1. The animals received 2x10^7^ PFU RhCMV-eGFP live or UV-inactivated cell-free virus (in DMEM+ 2% FBS and 0.2M sucrose), or media only (DMEM+ 2% FBS and 0.2M sucrose). A subset of four animals received an intravenous inoculation with 7x10^7^ PFU of RhCMV-eGFP cell-free virus (in DMEM+ 2% FBS) at week 23 (Fig. 1). The four animals included one UV-inactivated RhCMV-eGFP control animal (4M) and three media control animals (1M, 2M, 6M) (Table 1).

**Table 1:** Inoculation schedule.

		Inoculation		
		Week 0	Week 8	Week 23
Group	Animal Identification	Subcutaneous^a^	Subcutaneous^b^	Intravenous^c^
**RhCMV-eGFP**				
	7M	RhCMV-eGFP	RhCMV-eGFP	−
	8M	RhCMV-eGFP	RhCMV-eGFP	−
	9M	RhCMV-eGFP	RhCMV-eGFP	−
	10M	RhCMV-eGFP	Media control	−
	11M	RhCMV-eGFP	Media control	N/A
	12M	RhCMV-eGFP	RhCMV-eGFP	−
**UV-inactivated RhCMV-eGFP control**				
	4M	UV-inactivated RhCMV-eGFP	UV-inactivated RhCMV-eGFP	RhCMV-eGFP
	5M	UV-inactivated RhCMV-eGFP	UV-inactivated RhCMV-eGFP	−
**Media control**				
	1M	Media control	Media control	RhCMV-eGFP
	2M	Media control	Media control	RhCMV-eGFP
	3M	Media control	N/A	N/A
	6M	Media control	Media control	RhCMV-eGFP

^a^Animals were inoculated with 7x10^7^ PFU of RhCMV-eGFP or UV-inactivated RhCMV-eGFP, or media alone.

^b^All animals were boosted with 2x10^7^ PFU of RhCMV-eGFP or UV-inactivated RhCMV-eGFP, or media alone; with the exception of two RhCMV-eGFP animals (10M, 11M) that received media alone.

^c^A subset of control animals was given intravenous inoculation with 7x10^7^ PFU of RhCMV-eGFP.

N/A animals no longer in the study due to previous euthanasia.

### Sampling

Peripheral blood was collected into serum, EDTA (for DNA isolation and complete blood count), or sodium heparin vacutainer tubes (for PBMC isolation). Urine samples were catheter-collected or collected from cage pan in which the urine was filtered through a 0.45 μm filter. Bronchoalveolar lavage (BAL) samples were collected using paediatric fibre-optic bronchoscope according to routine procedure. The bronchoscope was inserted into a lobar bronchi and 10 ml of sterile saline was instilled and immediately recovered from the same lobe. Saliva was collected from the oral cavity using 5 ml sterile syringes. Oral swabs were collected along the lower lip, the gum line, and in the buccal pouch using sterile Starswab Multitrans Collection & Transport System swabs (Starplex Scientific Inc.). Genital samples were collected by inserting swabs under the prepuce. Immediately after sampling, the swabs were placed in tubes containing 1 ml DMEM (supplemented with 10% FBS, penicillin, streptomycin and amphotericin B). Four biopsies of the superficial inguinal lymph nodes were removed from the same site of inoculations using sterile surgical procedures, before and after inoculation.

### Sample Processing

Peripheral blood mononuclear cells (PBMCs) were isolated from whole blood by Percoll density gradient separation, as previously described [44]. Urine and BAL samples were centrifuged at 900 x g for 30 minutes at 4°C after which the supernatants were collected and mixed 1:1 with 2X MEM (supplemented with 2X antibiotic-antimycotic and 20 mg/ml gentamicin; Invitrogen). The cell pellets were resuspended with 1 mL of DMEM (supplemented with 1X antibiotic-antimycotic and 10 mg/ml gentamicin; Invitrogen). Centrifuged urine samples were further clarified by ultracentrifugation at 20,000 x g for 30 minutes at 4°C and the pellets were resuspended in 500 μl of DMEM (supplemented with 1X antibiotic-antimycotic and 10 mg/ml gentamicin). Saliva samples were mixed 1:1 with 2X MEM, and the oral and genital swabs were stored in transport medium. For DNA extraction, the oral and genital swabs were mixed with an equal volume of AL lysis buffer (Qiagen) and stored at -80°C. Inguinal lymph node biopsies from each leg were homogenized through a 100 μm cell strainer to isolate lymphocytes and centrifuged at 500 x g for 5 minutes at room temperature. The lymphocyte pellets were resuspended in R10 (RPMI Invitrogen; supplemented with 10% FBS, penicillin, streptomycin, L-glutamine) for viral cultures or resuspended in RPMI (+12.5% Human Serum Albumin) and 2X freezing media (RPMI + 10% HSA + 20% DMSO) for subsequent cryopreservation at -150°C.

### Immunophenotyping

Haematology (complete blood counts) and immunophenotyping were performed on a BD FACSCalibur and analysed using CELLQuest ProSoftware (BD Biosciences), as described previously [44,45]. Briefly, 5,000 CD45^+^ (clone TU116, BD Biosciences) lymphocytes were gated against low side scatter (SCC) and subsequently stained with lineage specific antibodies (BD Biosciences) to identify T cells [(CD3^+^ (SP34-2), CD4^+^ (L200), CD8^+^ (RPA-T8)], B cells [CD3^+^, CD20^+^ (2H7)] and NK cells [CD3^-^, CD16^+^ (3G8), CD56^+^ (MY31)]. To calculate the absolute counts/μl of blood, Flow-Count fluorospheres (Beckman Coulter) of known concentration were added to each sample and enumerated using the following equation: (total number of cell events counted/total number of Flow-Count bead events counted) x (Flow-Count assay concentration).

### Intracellular Cytokine Staining

Cryopreserved PBMCs were thawed, resuspended in R10 medium and plated in a V-bottom 96-well plate. Peptides (15-mers overlapping by 10 amino acids) spanning eGFP and RhCMV Immediate Early-1 (IE-1) protein were obtained from Mimotopes (Victoria, Australia). The peptides were individually dissolved in 80% dimethyl sulfoxide (DMSO) solution to 20 mg/mL and mixed to form peptide pools of 52 peptides for eGFP and 76 peptides for IE-1. The cells were stimulated with eGFP or IE-1 peptides (2.5 μg/mL/peptide) in the presence of antibodies to co-stimulatory molecules CD28 (CD28.2, NIH Reagent Resource) and CD49d (BD Pharmingen). Co-stimulation in the presence of staphylococcal enterotoxin B (SEB; Toxin Technology) or DMSO was used as positive and negative controls, respectively. After 2 hrs of incubation at 37°C and 5% CO_2_, monensin (5 μg/mL; Sigma) and brefeldin A (5 μg/mL; Sigma) were added to the wells and incubated for 6 hrs. Following overnight incubation at 4°C, the cells were stained with an amine-reactive live/dead stain (Invitrogen) for 30 min at 4°C. The cells were surface stained with conjugated mAbs to CD3 (SP34-2, BD Pharmingen), CD28 (CD28.2, eBioscience), CD95 (DX2, eBioscience), CD8 (RPA-T8, Invitrogen) and CD4 (L200, BD Horizon). Prior to intracellular staining, the cells were washed with FACS buffer, fixed and permeabilized using Cytofix/Cytoperm solution (BD Biosciences) for 20 min at 4°C. The cells were then stained with intracellular mAbs to IFNγ (B27, BD Pharmingen) and TNFα (MAb11, eBioscience) and acquired on a BD LSRII flow cytometer and the data was analyzed using FlowJo software (v. 9.2.6. TreeStar, Inc.). The values represent the percentage of total CD4^+^ and CD8^+^ T cell responses (background subtracted), and only responses greater than two-fold above background are shown.

### Viral co-culture

Human fetal lung fibroblast (MRC-5) cells [46] were obtained from the American Type Culture Collection (ATCC) and seeded 1:2 in a 12-well tissue culture plate 2 days prior to inoculation. The samples (urine, BAL, saliva, oral swabs, genital swabs and lymph nodes) were each plated in triplicate wells of MRC-5 cells in a 12-well tissue culture plate, with the exception of the ultracentrifuged urine samples which were added to a single well. Subsequently, the plates were centrifuged at 2,000 x g for 30 minutes at 4°C, and incubated for 2–3 hrs at 37°C in a 5% CO_2_ incubator. Following the incubation, the inoculum was aspirated and 2 ml of DMEM+10%FBS was added. The medium was changed the following day and monitored daily for CMV cytopathic effect. Urine samples were cultured from weeks 1–31, 71–81, and 201–206 post-inoculation, while the BAL, saliva, oral swab and genital swab samples were cultured from week 1 to week 16 and the lymph nodes were cultured at week 1 and 2 post-inoculation.

### Real-time quantitative PCR (qPCR)

Primers and probes were designed to amplify products specific for RhCMV 68–1 glycoprotein B (F:5’CACGGTTTTCTCCAAAATGC3’, R:5’GACTTCTGATGGTAAAGTTGTGGA3’, P:FAM-ATT GTTAGATCCATTGTAAAAAGGAGA-TAMRA), CyCMV glycoprotein B (F:5’CACCAAAATGTTTTCCGTTG3’, R:5’ATCGTCTGGTGATGTGGTG3’, P:FAM-CTGCCGTTGTAAAAGGGAGAT–TAMRA), and eGFP (F:5’ AACTACAACAGCCACAACGTCT3’, R:5’CGGATCTTGAAGTTCACCTTGAT3’, P:FAM-TATCATGGCCGACAAGCAGAAGAACG-TAMRA). DNA was extracted from whole blood, plasma, whole urine, urine pellet, saliva pellet and BAL pellet using a NucliSENS easyMAG instrument (bioMerieux) as per manufacturer’s protocol. Tissue culture-derived urine and lymph node biopsy DNA was extracted using DNeasy Blood and Tissue Kit (Qiagen) as per manufacturer’s protocol. Each reaction consisted of 2X QuantiTect PCR mastermix (Qiagen), primers (400 nM final each), probe (100 nM final), 5 μl of extracted DNA and water to a final volume of 50 μl. The real-time qPCR was run on an ABI Prism 7000 Sequence Detection System (Applied Biosystems) and the conditions were as follows: 1 cycle at 95°C for 10 minutes followed by 50 cycles at 95°C for 15 seconds and 60°C for 1 minute. Serial dilutions of the standard curve plasmid were prepared to quantitate the number of copies/ml and the results were analysed using SDS v1.2 software.

### Western Blot

Following six weeks of urine co-culture, the viral infection was graded in a blind fashion according to CMV CPE with a scale of: 1+ (0–20% CPE), 2+ (20–50% CPE), 3+ (50–80% CPE), 4+ (80–100% CPE), and the urine co-cultures were harvested. The cultures were pelleted by centrifugation at 15,300 x g for 10 minutes, washed with PBS and pelleted again. The pellet was resuspended in protease inhibitor cocktail (Sigma) and RIPA buffer (50 mM Tris HCl pH 8.0, 150 mM NaCl, 1% NP-40, 0.5% sodium deoxycholate, 0.1% SDS, 1 mM EDTA; adjusted to pH 7.4), and incubated on ice for 30 minutes. The lysate was centrifuged at 14,000 x g for 20 minutes at 4°C and the protein was quantitated using a Bio-Rad protein assay kit (Bio-Rad). Primary antibodies for western blotting included mouse anti-GFP (clone: BV-F4, 1:1,000, BioVision), mouse anti-RhCMV IE-1 (clone: 2A1.2, 1:750) kindly provided by Drs. Scott Hansen and Louis Picker (Oregon Health & Science University), and mouse anti-β actin (clone: AC15, 1:1,000; Sigma) as a lysate control. After probing with goat anti-mouse IgG (1:10,000, Cedarlane), the membrane was treated with Luminata Cresendo Western HRP substrate (Millipore) and exposed on an Amersham Hyperfilm ECL film (GE Healthcare).

### Enzyme-linked immunosorbent assay (ELISA)

96-well polystyrene plates (Corning) were coated with 0.15 μg/well of recombinant eGFP (Biovision) and blocked with 5% normal goat serum. Serum samples were added at a 1:40 dilution in duplicate, detected with peroxidase-conjugated goat anti-monkey IgG antibody (MP Biochemical) and developed with an ABTS HRP substrate (Thermo Scientific). Optical density (OD) values were determined using a Thermo Max Microplate Reader (Molecular Devices). Two or three replicate assays were conducted across all time points. Background OD values were subtracted and all time points were normalized to baseline (week -3) values.

### Illumina sequencing

Sequencing of RhCMV-eGFP was carried out at the University of Toronto (Department of Immunology) Sequencing Core Facility on an Illumina MiSeq desktop sequencer generating 150 bp paired end short reads with ~800X coverage. Raw Illumina reads were analyzed and assembled using Geneious Pro 6.1.4 (Biomatters Ltd., Auckland, New Zealand). Paired reads were trimmed at each end to ensure removal of any indexed tags. The reads were mapped to the expected sequence, virtually generated using the parental RhCMV 68–1 BAC sequence (GenBank Accession No. JQ795930) since the RhCMV-eGFP was derived from the RhCMV 68–1 BAC [47] [41] in Geneious Pro 6.1.4, using Burrows-Wheeler Aligner algorithm [48] to confirm the integrity of the virus and the site of insertion of eGFP. In addition, the reads were also mapped to RhCMV 68–1 genome sequence (GenBank Accession No. AY186194) Variants were identified using a minimum variant frequency of 0.25 and minimum coverage of 5. Gene boundaries were determined by alignment with homologous regions of previously published CMV genomes. Genome annotation was transferred from RhCMV 68–1 sequence (GenBank Accession Numbers: AY186194 or JQ795930).

### Statistical Analysis

A Kruskal-Wallis test was performed to compare mean difference between all groups, followed by a Mann-Whitney test for post-hoc analysis of individual group differences. To examine changes between time points, all time points were compared to baseline using a Wilcoxin test. Statistical analysis was performed using SPSS for Macintosh (Rel. 20.0.0. 2011. SPSS Inc.).

## Results

### Clinical outcomes and immunophenotyping

**Fig 2:**
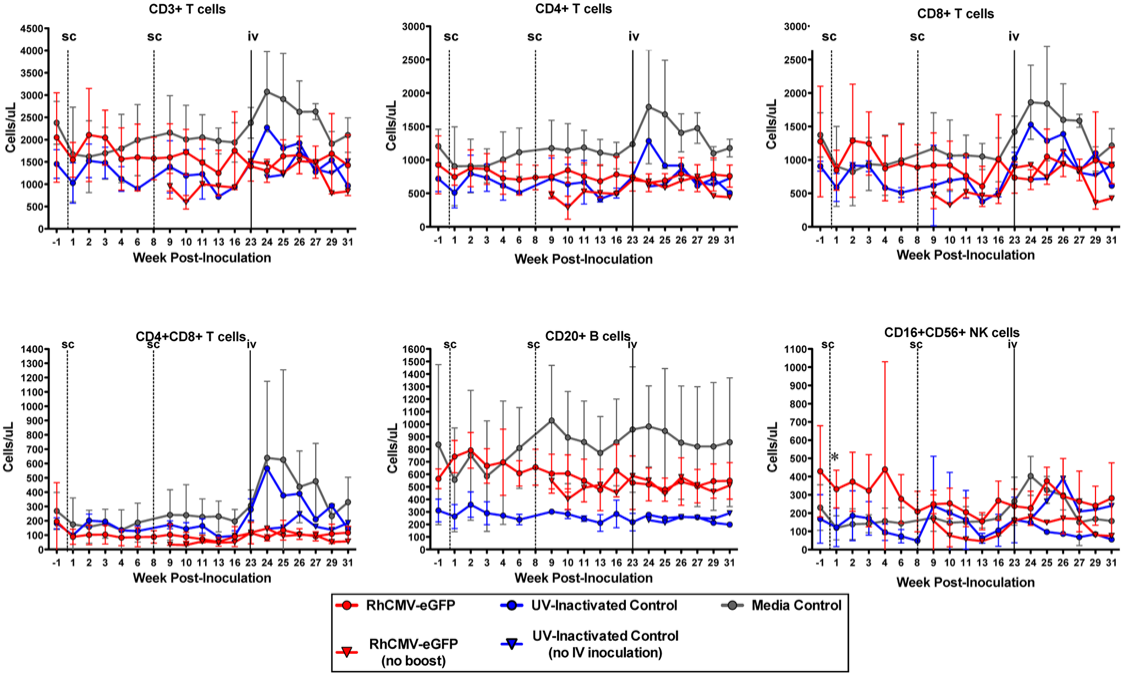
Immunophenotyping. Absolute values (cells/μl of blood) of T cell subsets (CD3+, CD4+, CD8+, CD4+CD8+), B cells (CD20+) and NK cells (CD16+CD56+) were determined by Flow Cytometry. Each point represents the mean of the animals in the group with error bars showing the standard deviation. The dashed vertical lines (<week 1 and week 8) represent subcutaneous inoculations and the solid vertical line (week 23) represents intravenous RhCMV-eGFP inoculations. The lines represent the same animals that were followed up to week 31 and the animals within each group are as follows: RhCMV-eGFP (n = 6, weeks 1–8;n = 4, weeks 9–23; n = 4, weeks 24–31); UV-inactivated group (n = 2, weeks 1–8; n = 2, weeks 9–23; n = 1, weeks 24–31); media control (n = 4 [1 up to week 4], weeks 1–8;n = 3, weeks 9–23; n = 3, weeks 24–31). The inverted arrow (◉) refers to the mean values from the animals in RhCMV-eGFP (n = 1, weeks 24–31) and UV-inactivated without IV inoculation (n = 1, weeks 24–31). * denotes a statistically significant difference (p = 0.01) between the RhCMV-eGFP and media control group.

At the study onset, all animals were healthy with comparable body weights and immunophenotyping profiles. Following inoculations, none of the animals displayed any clinical symptoms consistent with viral infections such as fever or loss of appetite throughout the course of the study. Animals 3M and 11M were euthanized at weeks 4 and 10 post-inoculation, respectively, due to progressive disease unresponsive to medical and surgical management caused by obstructive urosepsis and scrotal cellulitis respectively. In both cases, the decision to euthanize was made by two veterinarians who felt it was in the best and most humane interest of the animals and to ensure the animal would not suffer, and at no time was death considered an acceptable endpoint. Euthanasia of animals 3M and 11M, was conducted initially by deep sedation with ketamine (10 mg/kg i.m.), and then animals were transferred to a biosafety level II necropsy room. The animals were then deeply anesthetized with isofluorane and euthanized by aortic exsanguination inside a class II biosafety cabinet. At the end of the study, the remaining animals were recycled for use in unrelated ongoing studies. Animals had large single cages exceeding the minimum requirements with areas of both privacy and visual social interaction with other animals. Animals were given a daily comprehensive program of environmental enrichment to prevent abnormal behavior and minimize stress. Changes in body weight were monitored by calculating the percent initial body weight relative to baseline (week -1), and all animals showed an increase in body weight over four years with no significant difference between groups. To monitor the general health status of the animals and enumerate lymphocyte populations, complete blood counts comprising 20 haematology markers, and immunophenotyping were performed. The haematology results did not reveal any abnormalities in the animals and no significant differences were observed between the groups (data not shown). Examination of T cell (CD3^+^, CD4^+^, CD8^+^, CD4^+^CD8^+^), B cell (CD3^+^CD20^+^) and NK cell (CD3^-^CD16^+^CD56^+^) populations showed typical fluctuations in lymphocyte counts with no clinically significant changes in the absolute number (Fig. 2) or percentage (data not shown) of immune cells within or between groups following any of the three inoculations with RhCMV-eGFP. Though it did not reach statistical significance, at week 1 post-inoculation the absolute (cells/μl) and percentage (%) of NK cells was higher in the RhCMV-eGFP group [mean 332 cells/μl; 12.81%] compared to the media control group [mean 119 cells/μl; 5.4%; p = 0.01] and RhCMV-eGFP UV-inactivated control group [mean 122 cells/μl; 7.7%). It should be noted that at baseline (week -1), there was a trend towards an increased number of NK cells in the RhCMV-eGFP group (mean 429 cells/μl; 13.48%) relative to both the media (mean 230 cells/μl; 6.52%) and UV-inactivated (mean 168 cells/μl; 8.05%) control groups. Furthermore, a trend towards increased lymphocyte counts was observed in the four animals that received the intravenous inoculation with RhCMV-eGFP at week 23 (Fig. 2), however it was not statistically or clinically significant.

### eGFP and CMV IE-1-specific responses

To examine cellular immune responses to eGFP and IE-1, intracellular cytokine staining was performed on PBMCs isolated at baseline (week -1) and following the three inoculations (weeks 6, 13, and 29). PBMCs were stimulated with peptides spanning eGFP or CMV IE-1 protein (homologous between RhCMV and CyCMV), and stained with IFNγ and TNFα to examine the percentage of responding CD8^+^ and CD4^+^ T cells. Only responses that were two times the background response were included in the analysis. While no eGFP- specific responses were observed at baseline in any of the animals (Table 2A), UV-inactivated RhCMV-eGFP animals and one media control animal showed IE-1-specific responses at baseline (Table 2B). In total, five animals (3 RhCMV-eGFP, 2 media control) exhibited eGFP-specific CD8^+^ and/or CD4^+^ T cell responses following the subcutaneous inoculations (week 6 and week 13) (Table 2A). Immune responses to eGFP in the media control animals (2M and 6M) were also observed suggesting that these responses in both the groups were not specific to eGFP. The IE-1 peptides were designed to span a region of the IE-1 ORF that was homologous between RhCMV and CyCMV, thus we would expect cross-reactive T cell responses, and furthermore that these responses would be augmented if the CyCMV-seropositive animals were productively infected with RhCMV-eGFP. IE-1-specific CD4^+^ T cell responses were observed following the first subcutaneous inoculation (week 6) in 3 RhCMV-eGFP animals (9M,10M,12M) and one media control animal (6M), as well as following the second subcutaneous inoculation (week 13) in one UV-inactivated RhCMV-eGFP control animal (5M) (Table 2A). Two animals had IE-1-specific CD8^+^ T cell responses following RhCMV-eGFP inoculation by the subcutaneous (week 6: 12M) or intravenous (week 29: 1M) route (Table 2B).

**Table 2:** Intracellular cytokine staining of eGFP and IE-1 stimulated PBMCs.

(A) eGFP-specific T cell responses^a^																
CD8+									CD4+							
IFNγ					TNFα				IFNγ				TNFα			
Week	-1	6	13	29	-1	6	13	29	-1	6	13	29	-1	6	13	29
**RhCMV-eGFP**																
7M	−	−	0.91	−	−	−	0.64	−	−	−	−	−	−	0.02	0.01	−
8M	−	−	−	−	−	−	−	−	−	**0.01**	−	−	0.14	−	0.08	−
9M	−	−	0.40	0.06	−	0.01	0.21	−	−	**0.01**	**0.28**	−	−	**0.03**	0.04	0.01
10M	−	−	0.91	0.27	−	−	0.45	0.44	0.02	0.08	−	−	−	−	0.22	0.05
12M	−	**0.04**	0.22	−	−	**0.04**	−	−	−	**0.03**	0.06	0.08	−	0.08	0.03	0.03
**UV-inactivated RhCMV-eGFP control**																
4M	−	−	−	0.03	−	−	−	0.03	−	−	−	−	−	−	−	0.08
5M	−	−	0.51	−	−	−	0.39	0.14	−	−	0.04	−	−	−	0.17	0.12
**Media Control**																
1M	−	−	−	0.03	−	−	−	0.02	−	−	−	−	−	−	0.13	−
2M	−	**0.01**	−	−	−	−	−	−	−	−	−	0.03	−	−	−	−
6M	−	0.06	0.25	−	−	0.07	0.33	−	0.05	0.03	**1.37**	−	−	0.02	0.10	−
**(B) IE-1-specific T cell responses** ^a^																
**CD8** ^**+**^									**CD4** ^**+**^							
**IFNγ**					**TNFα**				**IFNγ**				**TNFα**			
**Week**	**-1**	**6**	**13**	**29**	**-1**	**6**	**13**	**29**	**-1**	**6**	**13**	**29**	**-1**	**6**	**13**	**29**
**RhCMV-eGFP**																
7M	−	−	−	−	−	−	−	−	−	−	−	0.04	−	−	−	−
8M	−	−	−	−	0.02	−	−	−	−	−	−	−	0.02	−	−	−
9M	−	−	−	−	−	−	−	−	−	**0.01**	0.01	−	−	**0.07**	−	0.14
10M	−	−	−	−	0.05	−	−	0.17	−	**0.18**	−	−	−	−	−	0.26
12M	−	**0.09**	−	0.01	−	**0.03**	−	−	0.03	**0.08**	0.01	0.09	−	**0.11**	−	−
**UV-inactivated RhCMV-eGFP control**																
4M	0.01	−	0.04	0.01	−	−	−	0.03	0.12	−	−	0.09	0.01	0.08	0.12	0.10
5M	0.26	−	2.02	0.07	0.05	−	1.38	−	0.02	−	**0.31**	−	−	−	0.03	−
**Media Control**																
1M	−	−	0.20	**0.07**	−	−	−	0.05	−	−	0.25	−	−	0.01	0.18	−
2M	−	−	−	−	−	0.01	−	0.01	0.03	−	−	0.01	−	−	−	−
6M	0.03	0.08	−	−	−	0.08	−	−	−	**0.01**	−	−	−	−	−	0.02

^a^The values represent the percentage of total CD8^+^ and CD4^+^ T cell responses (background subtracted). Following background subtraction, the negative or zero values were annotated with a dash (-) and the responses that were greater than two times the background are highlighted in bold.

### Viral co-culture screening for RhCMV-eGFP expression

Samples from easily accessible sites of CMV infection and routine shedding (including urine, BAL, saliva, oral and genital swabs, lymphocytes) were cultured on MRC-5 cells to screen for concurrent eGFP expression and CMV cytopathic effect (CMV-CPE), which is characterized by the presence of enlarged rounded cells in the form of clusters. eGFP expression was not observed in any of the tissue co-cultures throughout the course of the study. CMV CPE was observed in the urine and saliva cultures of 11 animals, with only one animal, 9M, having no detectable CMV CPE in any of the 23 samples cultured throughout the course of the study during which urine and saliva samples were collected from weeks 1 to 16 and only urine samples were collected from weeks 24 to 31 and later at weeks 71, 73, 75, 77, 79 and 81 post-inoculation. Due to the high prevalence of endogenous CyCMV in the animals from this colony [29], we would expect CyCMV to be shed in these cultures. The majority of the saliva-inoculated cultures displayed CPE typical of simian foamy virus (SFV), which is also routinely shed in the saliva of SFV-seropositive cynomolgus macaques [49] and is characterized by a vacuolizing “foam-like” effect on the cells [50]. SFV infection in the saliva co-cultures precluded the visualization of CMV CPE, and as such only the urine tissue cultured samples were harvested for CMV strain analysis. Total DNA was isolated from the urine cultures of two RhCMV-eGFP animals and two media control animals for phylogenetic assessment by PCR amplification of the highly conserved glycoprotein B (gB) gene, as previously described [29]. The resulting PCR products were sub-cloned and sequenced by Sanger sequencing with the resultant gB sequences from the four animals sharing 99.9% identity at the nucleotide level. The nucleotide sequences from the isolated samples were aligned with RhCMV 68–1 gB [27] and only 86% nucleotide identity was observed, suggesting that the CMV being shed in the urine of these animals was pre-existing CyCMV and not from productive RhCMV-eGFP infection.

In addition, urine samples from a select group of 8 animals (1M, 2M, 5M, 7M, 8M, 9M,10M and 12M) were collected biweekly at later time points from week 71 to week 81 post-inoculation and cultured on MRC-5 cells. Similar to the previous time points examined, CMV-specific CPE was observed in 7 of the 8 animals (with the exception of 9M) however, no eGFP expression was seen in any of the co-cultures. Following the completion of this study, five animals (1M, 7M, 8M, 9M, 10M) were subsequently enrolled into an SIV vaccine trial (Willer, D. O. et al., unpublished). Previous studies have shown that SIV infection in RhCMV-seropositive rhesus macaques leads to reactivation of latent RhCMV [51]. In an effort to determine if RhCMV-eGFP could be detected in these animals following SIVmac239 infection, urine samples were collected weekly from week 201 to 206 week post-RhCMV-eGFP inoculation and cultured for 6 weeks on MRC-5 cells. Similar to the earlier time points, CMV CPE was visible in all urine co-cultures, however eGFP expression was not observed. In 9 of the 12 animals, with the exception of 1M, 2M and 11M, eGFP-negative CMV CPE could be observed in the BAL co-cultured cells at week 1 post-inoculation with no subsequent detection thereafter. CPE was also detected in oral swabs of three RhCMV-eGFP animals at various weeks [8M (weeks 3, 8, 9, 11), 11M (weeks 4, 9), 12M (weeks 2, 3, 8, 9, 11)] and in the two UV-inactivated RhCMV-eGFP animals (4M, 5M) at week 1, however in no instance did we observe CPE-expressing eGFP. In the genital swab cultures, eGFP-negative CPE was detected in two of the six RhCMV-eGFP animals [8M (week 9), 11M (week 4)] and at week 1 in the two UV-inactivated RhCMV-eGFP animals (4M, 5M).

### Detection of RhCMV-eGFP by qPCR

Real-time quantitative polymerase chain reaction (qPCR) assays specific for CyCMV gB, RhCMV gB, and eGFP were used to detect and differentiate between the endogenous (CyCMV) and the inoculating strain (RhCMV-eGFP). Over the course of the study, an exhaustive number of real-time qPCR assays were performed in whole blood, plasma, urine (whole urine, urine pellet, and DNA-derived from urine co-cultures), BAL, saliva, and lymph node biopsies (Table 3). In all, 3,233 PCR reactions were conducted over the course of 4 years post-inoculation in an attempt to amplify RhCMV-eGFP-specific products and delineate between endogenous CyCMV and potential RhCMV-eGFP infection. CyCMV gB was detected in the inguinal lymph node biopsies acutely following primary inoculation in 33% of the animals. At week 4 post-inoculation, CyCMV gB was detected in the DNA-derived from urine co-cultures in animal 9M and acutely following the second inoculation in 5 animals: 8M, 9M, 11M (RhCMV-eGFP), 5M (UV-inactivated RhCMV-eGFP control), and 6M (media control). As previously determined by ELISA, all these animals were CyCMV-seropositive, however based on the results of the CyCMV gB qPCR assay, CyCMV was either not being persistently shed or the assay was not specific enough to detect CyCMV gB in these samples.

**Table 3:** Number of real-time qPCR assays.

Antigens tested (CyCMV gB: RhCMV gB: eGFP)								
	Whole Blood	Plasma	Whole Urine	Urine Pellet^a^	Co-cultured Urine	Saliva Pellet	BAL Pellet	Lymph Nodes
**RhCMV-eGFP**								
7M	18:19:19	16:15:15	16:16:16	20:15:20	3:07:07	11:11:11	11:11:11	4:04:04
8M	18:19:19	16:15:15	17:17:17	18:18:18	3:09:09	11:11:11	11:11:11	4:04:04
9M	20:19:19	17:16:16	17:17:17	20:20:20	3:09:09	11:11:11	11:11:11	4:04:04
10M	20:19:19	17:16:16	17:17:17	22:22:22	3:09:09	11:11:11	11:11:07	4:04:04
11M	9:08:08	6:05:05	5:05:05	5:05:05	2:02:02	7:07:07	7:07:07	4:04:04
12M	20:19:19	17:16:16	17:17:17	21:21:21	2:03:03	11:10:09	11:11:11	4:04:04
**UV-inactivated RhCMV-eGFP control**								
4M	19:18:18	17:16:16	18:18:18	24:24:24	2:02:02	11:11:11	11:11:11	4:04:04
5M	19:18:18	16:15:15	17:17:17	22:22:22	2:08:08	11:11:11	11:11:11	4:04:04
**Media control**								
1M	19:18:18	17:16:15	16:16:15	19:20:18	3:07:07	11:11:11	11:11:11	2:02:02
2M	19:18:18	17:16:16	18:18:18	21:21:21	2:04:04	11:11:11	10:10:10	4:04:04
3M	6:05:05	3:02:02	2:02:02	3:03:03	1:01:01	4:04:04	4:04:04	4:04:04
6M	19:18:18	11:15:15	15:15:15	20:20:20	2:02:02	10:10:10	11:11:11	4:04:04

^a^Urine pellet or ultracentrifuged urine pellet

RhCMV gB could not be detected by PCR under any circumstances whereas in the plasma and DNA-derived from urine co-cultures, non-specific eGFP PCR bands were detected. The PCR bands were observed with equal frequency in all groups, including the media control animals, which were only inoculated with media at the time point of detection suggesting that these bands were likely false positives. Following the first inoculation, two animals 12M (RhCMV-eGFP) and 3M (media control) had detectable eGFP in the plasma at 4400 copies/ml and 254 copies/ml, respectively. In the DNA derived from urine co-culture, the bands were sporadically detected at weeks 4, 6, and 9 post-inoculation in all animals, with the exception of 6M. Detectable quantities of eGFP were comparable across groups and ranged from 10^2^ to 10^4^ copies/DNA extraction with no evidence to suggest elevated copy numbers in the RhCMV-eGFP inoculated animals. Urine samples from 8 animals (excluding 3M, 4M, 6M and 11M) were cultured on MRC-5 cells at weeks 71, 73, 75, 77, 79 and 81 post-inoculation to determine if RhCMV gB and eGFP could be detected by qPCR at these later time-points. We were unable to detect RhCMV gB and eGFP in any of the animals at these time-points. Furthermore, at week 201 post-inoculation, DNA isolated from urine co-cultures of the five animals (1M, 7M, 8M, 9M, 10M) enrolled in an SIV vaccine trial and infected with SIVmac239 (Willer, D. O. et al., unpublished) was tested for detectable copies of eGFP. Similar to the previous results, only background quantities of eGFP were detected, as determined by the comparable level of detection in the animals (C02-003M, 21M) that were not in this RhCMV-eGFP inoculation study but were included as a control for SIV infection (data not shown).

### Detection of eGFP and CMV IE protein by Western blotting

**Fig 3:**
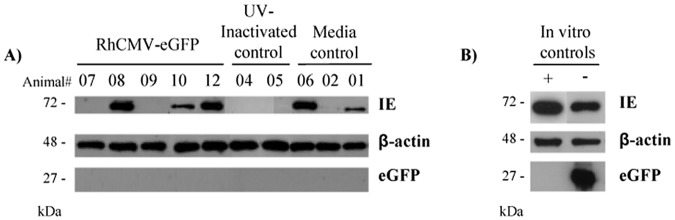
eGFP protein expression is not detected in RhCMV-eGFP inoculated cynomolgus macaques. Total protein was isolated from urine co-cultures in Telo-RF infected cells and probed by Western blot analyses with antibodies specific for eGFP (27 kDa), IE (72 kDa), and β-actin (42 kDa) as a lysate control. A) Analyses were performed following the two subcutaneous inoculations (week 16). Animals are listed by animal number as XX. B) In vitro infected Telo-RF cells were used for the eGFP controls with RhCMV-eGFP-infected cells as a positive control (+) and CyCMV_Ott-_infected cells as a negative control (-). Western blots from different experiments were combined for analyses.

Total protein, isolated from urine co-cultures displaying CMV CPE, was probed with anti-eGFP antibody to determine if eGFP protein could be detected. The eGFP gene in RhCMV-eGFP was under the control of the CMV immediate early (IE) promoter and thus would be expressed concomitant with the immediate-early genes. As an added control, an RhCMV IE antibody that cross-reacts with CyCMV-specific IE was included to demonstrate sufficient viral infection in the co-cultures at the time of isolation and was specific enough to detect the immediate-early transcribed proteins. Positive IE responses were observed in the CyCMV-seropositive media controls and in the CyCMV-infected in vitro control, providing further confirmation that the RhCMV IE antibody was cross-reactive with CyCMV-specific IE protein (Fig. 3). IE was detected only in viral co-cultures with greater than 2+ CPE (20–50% CPE) at the time of harvest. At week 16 post-inoculation, CPE in co-cultures from two RhCMV-eGFP animals (7M, 9M) and two UV-inactivated RhCMV-eGFP control animals (4M, 5M) was graded as 2+ or less, which was not sufficient to detect IE by Western blot however, one media control animal (2M) had 2+ CPE in culture and a faint IE response was observed (Fig. 3A). Following the two subcutaneous inoculations, we were unable to detect eGFP in any of the urine co-culture samples.

### Examining anti-eGFP antibody responses by ELISA

**Fig 4:**
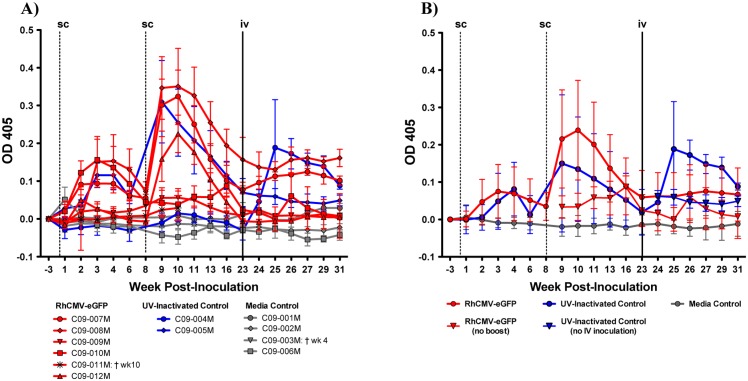
Anti-eGFP antibody responses present in RhCMV-eGFP and UV-inactivated RhCMV-eGFP control animals. Anti-eGFP antibody responses were determined by ELISA assay, values represented by optical density (OD). The mean of the replicate wells for each animal (A) or the mean of the animals in each group (B) are shown with error bars denoting the standard deviation. The dashed vertical lines represent subcutaneous inoculations and the solid vertical line represents intravenous RhCMV-eGFP inoculation of four control animals (1M, 2M, 4M, 6M). B) The mean from the animals in each group that were omitted from the second subcutaneous boost at week 8 (RhCMV-eGFP group) or the intravenous inoculation at week 23 (UV-inactivated group) (Table 1) are plotted with an inverted arrow (◉).

To determine if eGFP-specific antibodies were present in the serum of these animals, an eGFP-specific ELISA was performed. The UV-inactivated RhCMV-eGFP group served as a control for eGFP-specific antibody responses generated in response to RhCMV-eGFP proteins present in the inoculum, as opposed to antibodies produced as a result of replicating virus. There were no statistically significant differences between the groups across any time-points. In the RhCMV-eGFP inoculated group, 5 of the 6 animals (excluding 11M) had a primary anti-eGFP antibody response peaking at week 2 or 3 post-inoculation followed by an elevated memory response two weeks after the second exposure (week 10), with the exception of animals 10M and 11M that did not receive the subcutaneous boost (Fig. 4; Table 1). Likewise, in the UV-inactivated RhCMV-eGFP control group, all animals had detectable eGFP-specific antibodies suggesting that the antibodies were produced in response to the protein present in the inoculum and not in response to replicating RhCMV-eGFP virus. One animal (5M) had a primary antibody response at week 3 following the first subcutaneous inoculation with UV-inactivated RhCMV-eGFP and an augmented memory response at week 9; while the other animal (4M) did not have a primary response until after the subcutaneous boost peaking at week 10 followed by a secondary response to the intravenous inoculation with live RhCMV-eGFP, which peaked at week 25. None of the media control animals exhibited anti-eGFP antibody responses to the subcutaneous media inoculations. Following intravenous inoculation of the media control animals with RhCMV-eGFP at week 23, one animal (1M) had a primary response that reached its peak at week 29 (Fig. 4A). When examining intra-group changes in antibody responses across all time-points relative to baseline (week -3), the RhCMV-eGFP and UV-inactivated RhCMV-eGFP groups displayed comparable antibody response profiles (Fig. 4B).

### Confirmation of viral genome integrity

**Fig 5:**
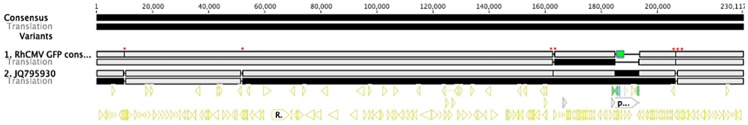
Sequence alignment comparing RhCMV-eGFP to RhCMV 68–1 BAC. Sequence of RhCMV-eGFP obtained from NGS analysis was aligned with that of RhCMV 68–1 BAC (JQ795930) using Geneious Pro 6.1.4 software. Whole genome comparisons yielded 96.2% similarity and the major difference can be attributed to the BAC cassette present in RhCMV68-1 BAC. (∎) refers to eGFP reporter * sequencing error;** 22 bp deletion; *** 56 bp insertion.

Next generation sequencing (NGS) of the RhCMV-eGFP genome resulted in a single contig of 224,626 bp. The eGFP coding region was inserted between Rh184 and Rh185 (186,659 bp). Compared to a predicted build based on the parental RhCMV 68–1 BAC strain from which RhCMV-eGFP was derived, as described in the methods section, our analysis show that, as expected, the pBelo11-derived BAC cassette was the only major difference between RhCMV-eGFP and the parental RhCMV 68–1 BAC (Fig. 5). In addition, there was one sequencing error in Rh64 and another in Rh10 in RhCMV-eGFP, a 56 bp insertion in the coding DNA sequences (CDS) of Rh199 in RhCMV-eGFP and the deletion of 22 bp between 162,600 and 162,622 of RhCMV-eGFP. However, when compared to the original RhCMV 68–1 viral genomic sequence, from which the parental BAC was derived, RhCMV-eGFP virus contained the expected mutations, frameshifts, deletions and insertions in their respective ORFs (S1 Table).

## Discussion

Superinfection with multiple strains of CMV has been well documented in HCMV-seropositive immunocompetent [6,52] and immunocompromised [9–11] individuals as well as in RhCMV-seropositive rhesus macaques [53,54]. It has been observed that greater than 95% of captive-bred NHPs are CMV-seropositive [55], which is comparable to our evaluation of the CyCMV-seroprevalence in the cynomolgus macaque colony at the Public Health Agency of Canada demonstrating close to 100% seropositivity [29]. At the time of this study, a CMV-free cynomologus macaque colony was not available and only recently have efforts been made to create CMV-free NHP colonies. Given that human and rhesus macaques are readily superinfected with their respective CMV strains, it is believed that superinfection in cynomolgus macaques is achievable. The inoculation dose used in this study was supported by previous studies, which demonstrated that RhCMV-seropositive rhesus macaques can be productively infected with a recombinant RhCMV 68–1 expressing a simian immunodeficiency virus (SIV) gag protein (RhCMV-gag) when given subcutaneously over a wide range of doses from as low as 10^2^ PFU and up to 10^7^ PFU, and furthermore the virus and exogenous protein could be detected through shedding in the urine and saliva [53,54]. Thus, our chosen dose of this exact strain represents an elevated dose and should have the capacity to infect cynomolgus macaques. The route of primary inoculation for the current study was chosen to most closely approximate the subcutaneous route of inoculation for CMV-based vaccines in humans. However, following two unsuccessful attempts to superinfect cynomolgus macaques with RhCMV-eGFP, we chose to pursue a potentially more robust route of inoculation by inoculating four control animals intravenously in an attempt to circumvent any pre-existing immune response. If pre-existing immunity to CyCMV indeed played a role in preventing RhCMV-eGFP infection in these animals, the robust intravenous inoculation should overcome any potential mucosal or peripheral immune barriers. Of note, only animal 9M persistently tested CyCMV-negative based on viral co-culture assays, although CyCMV gB was detected by qPCR. If the inability to overcome superinfection was a limiting factor in this study, animal 9M may have been readily infected with RhCMV-eGFP, however this was not observed.

Similar to humans, cytomegalovirus infection is asymptomatic in immunocompetent macaques with no sign of clinical abnormalities. The hematologic response to primary cytomegalovirus infection is highly variable and can be characterized by a mild to moderate leukocytosis in rhesus macaques [56]. Thus if the animals in this study were productively infected with RhCMV-eGFP, an acute inflammatory response with elevated lymphocytes would be expected. However, given that our study animals were seropositive for CyCMV, a dramatic lymphocyte response to the RhCMV-eGFP inoculum would not be expected. Moreover, we did anticipate that their CMV-specific lymphocytes would cross-react with the RhCMV antigens and thus generate an anamnestic response to the inoculum. The immunophenotyping results do not show any clinically significant variation between the groups following subcutaneous inoculations, with the exception of week 1 post-inoculation in which a non-significantly higher number and percentage of NK cells were observed in the RhCMV-eGFP animals compared to the media control animals. Following intravenous inoculation with RhCMV-eGFP, only animals in the control groups showed clinically insignificant increases in immune subsets (Fig. 2). Other studies have noted that intravenous inoculation with RhCMV in seronegative rhesus macaques led to an increase in absolute CD8^+^ T-cells and NK cells with no change in CD4^+^ T-cells at two weeks post-inoculation [56]. Our observations showed a modest increase in all cellular subsets one-week post-intravenous inoculation in the control animals, which was likely attributed to a systemic response to the serum (2% FBS) present in the inoculation.

Cytomegalovirus infection and viral reactivation is controlled by a robust cellular immune response [3–5]. The cell-mediated immune response to CMV infection in humans is generally characterized by an increase in IFN-γ secreting CD4^+^ T cells and the clonal expansion of CMV-specific CD8^+^ T-cells [3,5,57]. In RhCMV-seropositive rhesus macaques, a number of studies have demonstrated that the presence of pre-existing RhCMV-specific T cells does not prevent rhesus macaques from becoming infected with wild-type RhCMV or recombinant RhCMV-SIV [53,54,58]. Furthermore, it does not impede their ability to mount strong CD4^+^ and CD8^+^ T cell responses to RhCMV IE-1 [58] or to exogenous SIV antigens [53,54], and these responses increased over time. To examine cellular immunity following inoculation, we stimulated PBMCs with peptide pools spanning the eGFP or RhCMV IE-1 ORFs (Table 2). Modest CD8^+^ and CD4^+^ eGFP-specific T cell responses were observed in a subset of the RhCMV-eGFP animals and at comparable levels in a subset of control animals, thus the eGFP-specific responses were recognized as non-specific. The IE-1-specific T cell responses were sporadic and did not display any evidence for an increase in the percentage of responding cells following each inoculation and therefore the observed responses were most likely CyCMV-specific responses.

Primary RhCMV infection in the natural host results in detectable RhCMV 68–1 DNA as early as 2 weeks post-infection [56]. Similar to the broad tissue-tropism of HCMV, RhCMV infects multiple organs including the brain, liver, kidney, intestine, lungs, lymph nodes, spleen, pancreas, tonsils, salivary glands, bladder, thymus, bone marrow, plasma and blood [43,56,59]. As such, an extensive array of tissues was collected in this study in an attempt to detect RhCMV-eGFP DNA, protein, and infectious virus (Fig. 1). RhCMV-eGFP could not be detected in any of the samples using highly sensitive assays for CMV detection. This contrasts with studies performed by Hansen et al., where upon inoculation of rhesus macaques with RhCMV with a similar route and dose used in this study, it was shown that SIV proteins expressed in RhCMV vectors were readily detected by Western blot in urine and saliva co-cultures from RhCMV-seropositive rhesus macaques [54,60]. Thus, if productive infection occurred in our study it would have been detected over the course of the 31 weeks and undeniably at 4 years post-inoculation when a subset of animals were infected with simian immunodeficiency virus (SIV), which has been shown to stimulate latent CMV reactivation in macaques [51,61]. To determine if SIV infection would cause reactivation of latent RhCMV-eGFP, viral co-cultures were performed from urine samples of SIV-infected cynomolgus macaques. RhCMV-eGFP could not be detected in the co-cultures from any of the animals using the most sensitive assays for CMV detection, suggesting that RhCMV68-1 eGFP cannot productively infect cynomolgus macaques.

We have recently isolated, sequenced, and characterized the complete CyCMV_Ott_ genome and performed an extensive comparison between CyCMV, RhCMV, and HCMV [30,37]. CyCMV_Ott_ shares 89.8% nucleotide identity with RhCMV 68–1 and the genes are largely colinear; furthermore, of the 262 ORFs annotated in CyCMV_Ott_, 243 ORFs are homologous to RhCMV 68–1 [30,37]. Although whole genome alignment and phylogenetic analysis indicates that CyCMV_Ott_ is most closely related to RhCMV [30], RhCMV cannot overcome the species-specific barrier in cynomolgus macaques. The high nucleotide identity between CyCMV_Ott_ and RhCMV would suggest that the mechanism behind the strong species-specificity could be related to natural host range, although this has not been well elucidated. With the recent sequencing and documentation of the cynomolgus macaque genome [62,63], comparison between cynomolgus and rhesus macaque genomes may provide insight into host cellular restriction factors that may be playing a role in inhibiting cynomolgus macaques from being infected with RhCMV. Previous work has shown that HCMV has the ability to enter cells from a distant species, such as guinea pigs [64], however following entry, the infection is non-productive suggesting that the virus may induce the innate immune defence of the host cell thus preventing CMV replication. Furthermore, the restriction may be mediated by the inability of heterologous viruses from different species to overcome early post-entry steps, such as the host apoptotic defence [65]. Cytomegaloviruses encode anti-apoptotic genes (β2.7, UL36, UL37 ex1 and UL38) that function to overcome the host-mediated cellular apoptosis response following CMV infection [39]. The CyCMV homologues of the HCMV anti-apoptotic genes (cyUL36-cyUL38) share high homology (~96–97% identity) with their RhCMV homologues [30], however the results of this study suggests that it may not have been sufficient to prevent host-induced apoptosis. Although herpesviruses are notoriously species-specific, there have been studies in which these viruses have broken the species-specific barrier [45].

The infectivity of our RhCMV-eGFP clone was not confirmed in rhesus macaques due to unavailability of animals and remains a caveat of this study. Certainly to conduct a controlled nonhuman primate infectivity study in rhesus macaques confirming infectivity after any manipulation or in vitro passaging of a large herpesvirus such as RhCMV68.1 would have provided a definitive gold standard, the cost and logistics being a major limitation. A further limitation of infectivity studies is the requirement to consider the specific geographic origin of both the virus and animals and match them, particularly in the case of rhesus and cynomolgus macaques. Macaque interspecies hybridization has occurred in specific geographic areas of species overlap such as Vietnam [66] while greater genetic distance has been documented between the Indian rhesus macaque and the island populations of cynomolgus macaques such as the Philippines [67]. Cytomegalovirus isolated from the two species reflects this same progressive diversity and may be used as a surrogate to track the progressive genetic diversification of macaques [68](manuscript in preparation). Thus, it is our opinion that a careful documentation of the origin and genealogy of the host and virus be maintained and appreciated as highly significant since species are not absolute entities but are variably related entities and this has an impact on permissiveness to interspecies infection with pathogens.

To corroborate our findings, we performed extensive NGS analyses of the RhCMV-eGFP viral genome to determine its integrity and our results indicate that the changes observed in comparison to the parental RhCMV 68–1 BAC sequence were minimal and would not affect growth of the virus in tissue culture nor its ability to infect rhesus macaques (Fig. 5). During BAC cloning, a molecular clone of a selected virus strain is fixed as a genome and thus might differ in its genome content from the parental strain, which likely contains a mixture of molecular clones. As an example, the RhCMV 68–1 BAC is known to contain a mutation in Rh61/60, the RhCMV homologue of UL36, which is not present in RhCMV 68–1 sequence [69]. Additionally, the extensive in vitro propagation required for BAC cloning might select for additional tissue culture adaptations compared to the parental strain. To obviate these issues, Malouli et al., sequenced RhCMV 68–1 BAC by whole-genome shotgun sequencing and compared to the parental strain, several mutations in ORFs were found [47]. However, despite these mutations, the BAC-derived RhCMV was able to establish and maintain persistent infections upon experimental inoculation of CMV-naïve or CMV-positive rhesus macaques suggesting that persistent viral infection occurs despite multiple mutations [47].

In order to confirm the findings of Malouli et al., [47], the RhCMV-eGFP genome integrity was also compared to that of RhCMV 68–1 virus, which is known to successfully infect rhesus macaques. The virus contained 102 possible mutations and the vast majority of these were also present in the parental RhCMV 68–1 BAC from which our RhCMV-eGFP was derived (S1 Table). Moreover, we employed Illumina sequencing of RhCMV-eGFP whereas RhCMV 68–1 BAC was sequenced using a Roche 454 platform [47]. Comparative analysis point to high correlation between the two platforms but evaluation of base-call error, frameshift frequency and contig length suggest that Illumina offered equivalent, if not better, assemblies than Roche 454 [70]. It thus seems that the changes observed in RhCMV-eGFP will not alter its in vivo properties. In light of our findings in vitro, in vivo and in silico, a conclusion can be drawn that RhCMV-eGFP is viable but unable to infect cynomolgus macaques.

Since RhCMV 68–1 is a lab-adapted fibroblast-tropic virus strain, its inability to infect cynomolgus macaques might not be surprising. It is plausible that a wild-type rhesus CMV may be capable of infecting cynomolgus macaques but not the lab-adapted RhCMV 68–1 strain from which RhCMV-eGFP is derived. Though not directly analogous, the VZV-Oka vaccine strain that was passaged 23 times was not capable of infecting rhesus macaques [71] whereas the parental VZV-Oka strain that had been passaged 2 times was capable of doing so [45]. Our findings from the evaluation of RhCMV-eGFP infection in cynomolgus macaques suggest that this macaque species cannot be productively infected with RhCMV. Additional studies in CyCMV-naïve cynomolgus macaques may be more informative, however, based on previous RhCMV studies, CyCMV_-_serostatus should not be a confounding factor in the ability of RhCMV to overcome the species-specific barrier. Recent studies by Picker et al. have shown that using RhCMV as a viral vector for an SIV vaccine induces a strong effector memory immune response that is both durable and protective from SIV disease progression [54,60]. Furthermore, in macaques it was recently reported that in both vaccinated viral controllers and non-controllers, the majority of the RhCMV-SIV vectored effector memory CD8^+^ T cell responses were not directed towards the conventional MHC class I-restricted immunodominant and well-characterized SIV epitopes, but were in fact predominantly targeting non-conventional SIV epitopes that were MHC class II-restricted [72]. These findings demonstrate that the results of the RhCMV-SIV vaccine studies may be strain-specific as this epitope recognition phenomenon was unique to the fibroblast-adapted RhCMV 68–1 strain, which lacks the HCMV homologues of UL128-131 involved in cellular tropism. Hansen et al. propose that the absence of UL128-131, which would otherwise suppress US11, the MHC class I down regulation gene, mediates these unconventional MHC class II-restricted CD8^+^ T cell responses resulting in atypical epitope recognition [72]. However, the potential restriction of the cell tropism in vitro would classify RhCMV 68–1 as attenuated. Interestingly, CyCMV_Ott_ has retained the HCMV US128-131 homologues though it is a low-passaged virus that has adaptations to growth on fibroblasts [30]. Another unique difference between CyCMV_Ott_ and RhCMV is that CyCMV_Ott_ does not encode a viral cyclooxygenase-2 (COX-2) gene that appears to be unique to RhCMV [27,30], and in this regard makes CyCMV_Ott_ similar to HCMV. Now more than ever, CMV and a variety of CMV strains should be assessed as CMV-based HIV/SIV vaccine vectors before moving forward to human clinical trials. Moreover the differences between lab-adapted and primary CMV (parental strains) with respect to immune effector functions and species range, need to be considered. When the genetic factors that account for functional differences are identified, this information can be used to iteratively improve the functionality of nonhuman primate and HCMV vaccines for HIV or other important pathogens such as TB or Malaria.

## Supporting Information

S1 Checklist (PDF)

S1 Table Results of Next Generation Sequencing showing in detail all of the mutations, their location, frameshifts, deletions and insertions in their respective ORFs, their putative function and their effects on tissue culture growth in literature. Also provided is the observation made with respect to each ORF with their associated changes in tissue culture experiments in this study.—Information on ORF effect not available; +++ Good growth in Telo-RF cells; none refers to lack of HCMV homologue; Note: Variant frequencies above 50% shown
(DOC)
