# Correction: Examining the Species-Specificity of Rhesus Macaque Cytomegalovirus (RhCMV) in Cynomolgus Macaques

**DOI:** 10.1371/journal.pone.0128733

**Published:** 2015-05-14

**Authors:** The PLOS ONE Staff

There are multiple errors in Fig 5 and its caption, “Sequence alignment comparing RhCMV-eGFP to RhCMV 68–1 BAC.” Please see the complete, corrected Fig 5 here. The publisher apologizes for the error.

**Fig 5:**
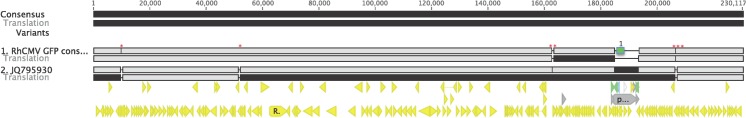
Sequence alignment comparing RhCMV-eGFP to RhCMV 68–1 BAC. Sequence of RhCMV-eGFP obtained from NGS analysis was aligned with that of RhCMV 68–1 BAC (JQ795930) using Geneious Pro 6.1.4 software. Whole genome comparisons yielded 96.2% similarity and the major difference can be attributed to the BAC cassette present in RhCMV68-1 BAC. “1” refers to eGFP reporter * sequencing error;** 22 bp deletion; *** 56 bp insertion.
