# Supplementary material for: Examining the Species-Specificity of Rhesus Macaque Cytomegalovirus (RhCMV) in Cynomolgus Macaques
Source: PLoS One. 2015 Mar 30;10(3):e0121339. doi: 10.1371/journal.pone.0121339 (PMC4378995; doi:10.1371/journal.pone.0121339)
Supplement: S1 Table — Also provided is the observation made with respect to each ORF with their associated changes in tissue culture experiments in this study.—Information on ORF effect not available; +++ Good growth in Telo-RF cells; none refers to lack of HCMV homologue; Note: Variant frequencies above 50% shown (DOC) [file pone.0121339.s002.doc]

**Supporting Information Table S1:** Results of Next Generation Sequencing showing in detail all of the mutations, their location, frameshifts, deletions and insertions in their respective ORFs, their putative function and their effects on tissue culture growth in literature. Also provided is the observation made with respect to each ORF with their associated changes in tissue culture experiments in this study.

- Information on ORF effect not available; +++ Good growth in Telo-RF cells; none refers to lack of HCMV homologue; Note: Variant frequencies above 50% shown
